# Clinical outcomes of individualized surgical pathways in elderly patients with spinal tuberculosis: a retrospective cohort study in eastern China

**DOI:** 10.3389/fmed.2026.1869144

**Published:** 2026-09-21

**Authors:** Bowen Han, Mingshan Zhang, Cheng Cui, Maolin Niu, Xue Liang, Zhixuan Li, Dapeng Wu, Xuejian Wu

**Affiliations:** 1 Department of Orthopedics, The First Affiliated Hospital of Zhengzhou University, Zhengzhou, Henan, China; 2 Department of Orthopedics, The First Affiliated Hospital of Henan Medical University, Xinxiang, Henan, China; 3 Xinxiang Key Laboratory of Spinal Infectious Diseases Research, Henan Medical University, Xinxiang, Henan, China; 4 Department of Orthopedics, Jiaozuo Second People’s Hospital, Jiaozuo, Henan, China; 5 Department of Orthopedics, Xiangcheng Orthopedic Trauma Hospital, Zhoukou, Henan, China

**Keywords:** elderly patients, individualized surgical pathway, recurrence, spinal tuberculosis, staged management

## Abstract

**Objective:**

Although surgical indications for adult spinal tuberculosis (TB) are well established, optimal surgical strategies for elderly patients remain uncertain. This study described the clinical outcomes of an individualized staged management pathway for elderly patients with spinal TB in eastern China and reported outcomes of a clinically distinct cohort undergoing combined posterior and anterior surgery. No comparative efficacy hypothesis was tested.

**Methods:**

This retrospective observational cohort study included patients treated between January 2020 and December 2022. Forty-two patients were classified according to treatment pathway. Group A comprised 23 patients managed with ultrasound-guided abscess drainage followed by posterior stabilization and posterolateral fusion, with anterior surgery reserved for inadequate infection control. Group B comprised 19 patients who underwent combined posterior and anterior surgery because immediate anterior debridement and/or interbody reconstruction were required. Because pathway selection was indication-based and the cohorts had different operative indications, outcomes were summarized descriptively rather than compared statistically. Index spinal operative time, index estimated blood loss, visual analog scale score, Oswestry Disability Index, kyphotic angle, Frankel grade, postoperative complications, and secondary surgery were recorded.

**Results:**

All patients achieved satisfactory clinical and radiographic outcomes during 24–45 months of follow-up. Mean index operative time was 211.26 ± 70.30 min in Group A and 315.68 ± 70.92 min in Group B, and mean index estimated blood loss was 406.52 ± 224.26 mL and 594.74 ± 139.33 mL, respectively. These values described different clinically selected index operations and were not statistically compared. At final follow-up, Frankel grade E was recorded in 19/23 (82.6%) patients in Group A and 16/19 (84.2%) in Group B, compared with 15/23 (65.2%) and 12/19 (63.2%) preoperatively. Recurrence occurred in one patient in Group A and three patients in Group B.

**Conclusions:**

The individualized staged pathway was completed without salvage anterior surgery in 22 of 23 Group A patients, while one patient required subsequent anterior debridement and interbody fusion. These descriptive data support feasibility in selected patients but do not establish that preoperative catheter drainage is necessary or superior to posterior surgery without drainage. Combined posterior-and-anterior surgery remained appropriate when immediate anterior debridement or reconstruction was indicated.

## 1 Introduction

Tuberculosis (TB) was the second leading cause of death from infectious diseases in 2022, following coronavirus disease (COVID-19), and it accounted for nearly twice as many deaths as human immunodeficiency virus/acquired immune deficiency syndrome, as reported in the World Health Organization Global Tuberculosis Report 2023 (1). Skeletal involvement occurs in 1%–2% of all TB cases, with spinal TB accounting for more than 50% of skeletal TB cases (2). Due to systemic factors such as malnutrition, diabetes, anticancer therapies, COVID-19, and the increased use of immunosuppressive medications, the incidence of spinal TB is rising among the elderly (3). Elderly patients may have compromised bone quality and general health, which can increase the risk of complications such as poor wound healing, implant failure, and infection when surgery is required. Additionally, elderly patients often have existing medical conditions and may take multiple drugs, making treatment more difficult.

Patients with spinal TB and disabling pain, progressive deformity, spinal instability, abscess formation, and late-onset neurological deficits may require surgery to remove infected tissue, stabilize the spine, and correct deformity (4). Various treatment options have been used for spinal TB, including combined anterior-plus-posterior, posterior, and anterior surgical approaches. Each strategy has benefits and drawbacks, and the choice of instrumentation depends on a number of variables, including the location and severity of spinal TB, the overall health of the patient, and the experience and preference of the surgeon. Currently, it is generally accepted that the posterior approach is less invasive, provides sufficient cord decompression and stronger three-column fixation, and may avoid complications associated with anterior exposure (5). It has been reported that preoperative percutaneous catheter drainage of paravertebral or psoas abscesses can reduce the abscess burden before definitive fixation, provide material for microbiological and pathological examinations, help improve systemic inflammatory status, and thereby reduce surgical trauma and postoperative complications (6). If the posterior elements and spinal stability can be adequately preserved or reconstructed, interbody bone grafting may not be necessary in selected patients (7).

However, surgical decision-making for elderly patients remains difficult because evidence and specific guidance regarding the most appropriate strategy are limited. Staged operations for spinal TB have been described previously (8, 9); therefore, the present study does not claim novelty for staging itself. At its first substantive mention, the individualized staged management pathway is defined as three potential components: (1) ultrasound-guided percutaneous catheter drainage as a preoperative interventional phase, (2) posterior fixation and posterolateral fusion with or without decompression as the definitive spinal operation, and (3) optional anterior debridement and interbody fusion as salvage when infection control remains inadequate. The timing and need for the later components were tailored to lesion morphology, neurological urgency, response to drainage, infection control, and the need for reconstruction. We describe the clinical outcomes of this pathway and report a clinically distinct cohort treated by combined posterior and anterior surgery for descriptive context only.

## 2 Material and Methods

### 2.1 Patients

This retrospective cohort study has been approved by the Ethics Committee of the First Affiliated Hospital of Zhengzhou University (Approval Number EC-024-004). All patients signed informed consent forms that contained details about the purpose of the procedure, surgical management, expected outcomes, possible risks, and side effects. This study was performed in accordance with the ethical standards of the 1964 Declaration of Helsinki and its later amendments. The diagnosis of spinal TB was based on clinical manifestations, medical history, laboratory examinations data, radiographic findings, and pathological analysis of the pus drained from the abscess (10). Some patients were initially evaluated or treated in primary medical centers or local hospitals and were subsequently referred to our institution for surgical management. After admission, all patients underwent standardized clinical, laboratory, and imaging assessments, and the diagnosis of spinal TB was confirmed before surgery.

### 2.2 Inclusion and exclusion criteria

The inclusion criteria were: (1) age ≥65 years, defined as elderly patients; (2) confirmed diagnosis of spinal tuberculosis based on clinical manifestations, laboratory tests, imaging findings, microbiological or pathological evidence when available, and response to anti-TB therapy; (3) surgical indications for Pott's disease, including persistent pain, spinal instability, progressive kyphotic deformity, neurological impairment, paravertebral or psoas abscess formation, or poor response to conservative treatment; (4) preoperative anti-TB therapy administered whenever possible, with surgery performed after systemic symptoms were controlled, except in patients requiring urgent surgical intervention because of progressive neurological deterioration or severe spinal cord compression; and (5) follow-up period of more than 24 months. The exclusion criteria were as follows: (1) poor general condition or severe systemic disease making surgery intolerable, corresponding to American Society of Anesthesiologists (ASA) physical status classification grade IV or higher after anesthesiology evaluation; (2) uncontrolled active pulmonary or systemic TB requiring systemic medical stabilization before spinal surgery; (3) mental illness or inability to communicate effectively; and (4) insufficient medical data.

Between January 2020 and December 2022, we retrospectively analyzed 48 elderly patients with spinal TB who underwent spinal surgery. Six patients lost to follow-up were excluded. The remaining 42 patients were classified according to the treatment pathway received: Group A (individualized staged management pathway, *n* = 23) and Group B (combined posterior-and-anterior surgery, *n* = 19). Selection was based primarily on lesion morphology, abscess accessibility and controllability, anterior-column integrity, neurological urgency, and the need for immediate anterior debridement or reconstruction. All patients met at least one prespecified surgical indication. Patient and family preferences were considered only after clinically appropriate options had been explained and were not the sole basis of pathway allocation (Supplementary Tables S1, S2).

### 2.3 Preoperative management

All patients received isoniazid, rifampicin, pyrazinamide, and ethambutol (HRZE) therapy (rifampicin, 15 mg/kg 600 mg/day; isoniazid, 5 mg/kg 300 mg/day; ethambutol, 15 mg/kg 750 mg/day; and pyrazinamide, 25 mg/kg 1500 mg/day) for more than 3 weeks before surgery whenever clinically feasible (11). For patients with progressive neurological deterioration, severe spinal cord compression, or rapidly worsening spinal instability, urgent surgery was performed after short-term intensive preoperative preparation, including anti-TB therapy, nutritional support, and correction of anemia or hypoalbuminemia when necessary (12). Surgery was generally scheduled after body temperature was <37.5 °C, systemic symptoms had improved, nutritional status had improved, and erythrocyte sedimentation rate (ESR) and/or C-reaction protein (CRP) showed a downward trend. Because this was a retrospective study, rigid laboratory thresholds were not uniformly applied; the decision to proceed with surgery was based on the combined assessment of symptoms, neurological status, imaging findings, inflammatory markers, nutritional status, and anesthesiology evaluation. Plain radiography, magnetic resonance imaging (MRI), and computed tomography (CT) were used to assess vertebral body collapse, osteopenia, endplate erosion, paravertebral or psoas abscess, epidural compression, and disc space destruction.

### 2.4 Outcome measurements and definitions

Sex, age, body mass index (BMI), symptoms, disease duration, underlying diseases, visual analog scale (VAS) score, Frankel grade, ESR, CRP level, Cobb's angle, involved segments, index spinal operative time, index estimated blood loss (EBL), transfusion, Oswestry Disability Index (ODI), complications, recurrence, secondary surgery, and follow-up duration were collected from the medical records. Disease duration was defined as the interval from symptom onset to admission and is reported in months. Index spinal operative time was defined as the interval from skin incision to wound closure for the definitive spinal operation, and index EBL referred to blood loss during that operation. In Group A, ultrasound-guided catheter drainage was a separate interventional procedure rather than a spinal operation. Its duration was not consistently documented in the retrospective operative and anesthesia records, and blood loss was not quantitatively recorded; therefore, these values could not be included. For the single Group A patient who later underwent salvage anterior debridement and interbody fusion, the operative time and EBL of that later procedure were recorded as a separate secondary operation and were not included in the index-operation means reported in Table 2. In Group B, index operative time included repositioning and preparation for the anterior phase because the posterior and anterior procedures were performed during the same anesthetic session. The reported operative-time and EBL values therefore describe the index spinal procedures and do not represent total treatment duration or cumulative procedural burden. Postoperative variables were recorded during the early postoperative hospitalization or before discharge unless otherwise specified, and final follow-up variables were recorded at the last outpatient visit. The study was prepared with reference to the Strengthening the Reporting of Observational Studies in Epidemiology (STROBE) recommendations for observational studies where applicable (13).

Neurological function was evaluated using the Frankel grading system (14). Although the American Spinal Injury Association (ASIA) Impairment Scale (AIS), incorporated in the International Standards for Neurological Classification of Spinal Cord Injury, is now more widely used for standardized neurological classification (15), Frankel grading was used in this retrospective study because it was the neurological classification consistently recorded in the medical records during the study period.

### 2.5 Operative techniques

The choice of pathway was indication-based rather than randomized. Within Group A, the term “individualized” refers to tailoring the timing of posterior surgery, the need for neural decompression, and escalation to salvage anterior surgery according to lesion morphology, neurological status, response to drainage, infection control, and the need for reconstruction; it does not indicate unrestricted patient preference. Group A (individualized staged management pathway) was selected when an accessible paravertebral or psoas abscess could be initially managed by percutaneous drainage and spinal stability could be restored through posterior fixation and posterolateral fusion, with or without decompression, without an immediate need for anterior-column reconstruction. Progressive neurological deterioration, severe spinal cord compression requiring immediate decompression, severe anterior-column destruction requiring reconstruction, an abscess not amenable to or controllable by percutaneous drainage, or concern that delay would compromise lesion control were considered contraindications to this pathway. Age or frailty alone was not an indication for drainage-first treatment. Within Group A, anterior debridement and interbody fusion were reserved as salvage for persistent or recurrent abscess, sinus formation, progressive vertebral destruction, poor infection control, or recurrence during follow-up. Group B (combined posterior-and-anterior surgery) was selected when radical anterior debridement and interbody reconstruction were considered necessary at the initial decision point, including severe anterior-column destruction, large abscesses unlikely to be controlled by drainage alone, marked disc or endplate destruction, or concern regarding insufficient lesion control using posterior surgery alone. Because the cohorts had different operative indications, they are described in parallel rather than treated as exchangeable comparison groups. Pedicle screws were generally inserted into uninvolved vertebrae one or two levels above and below the lesion. Screws were not inserted into vertebrae with severe destruction, obvious osteolysis, or poor pedicle integrity. In selected cases with relatively preserved pedicles and adequate bone quality, fixation including the affected vertebra was considered to enhance construct stability.

#### 2.5.1 Group A: individualized staged management pathway

All patients in this group underwent a preoperative interventional phase of ultrasound-guided percutaneous catheter drainage of paravertebral or psoas abscesses identified on imaging. A puncture needle was inserted into the abscess or lesion under ultrasound guidance. Typical lesion tissue and pus were obtained for mycobacterial culture, drug-sensitivity testing, and pathological examination when available, after which the drainage tube was connected to a vacuum drainage device. The drainage-first pathway was limited to patients without progressive neurological deterioration or severe cord compression requiring urgent decompression at the initial decision point. Such neurological urgency was a contraindication to Group A and required prompt surgical decompression rather than waiting for a drainage-output threshold. If neurological status worsened during drainage, posterior decompression was advanced without waiting for the drainage-output threshold. In Group A patients without an indication for urgent decompression, posterior surgery was performed when drainage output had decreased to <10 mL/day and systemic inflammatory status had improved. The exact drainage-to-surgery interval was not a prespecified variable and was not uniformly retrievable from the retrospective records. For the definitive posterior operation, patients were placed in the prone position under general anesthesia. An incision was made posteriorly along the midline, centering on the affected vertebral body. Following standard exposure of the posterior spinal structures, pedicle screws were inserted one or two levels above and below the lesion. Depending on MRI findings and neurological symptoms, decompression was performed when neural compression was present. Posterolateral fusion was performed in all patients. Based on follow-up radiological findings and symptoms, optional salvage anterior debridement and interbody fusion was reserved for persistent or recurrent abscess, sinus formation, progressive vertebral destruction, poor infection control, or recurrence. Epidural abscesses or compressive lesions causing neurological impairment were addressed by posterior decompression during the definitive spinal operation rather than by percutaneous drainage alone. The term staged management refers to this sequence of a preoperative interventional adjunct and one or more surgical phases; ultrasound-guided drainage was not considered a mandatory spinal surgical stage. The management pathway is illustrated in Figure 1.

**Figure 1:**
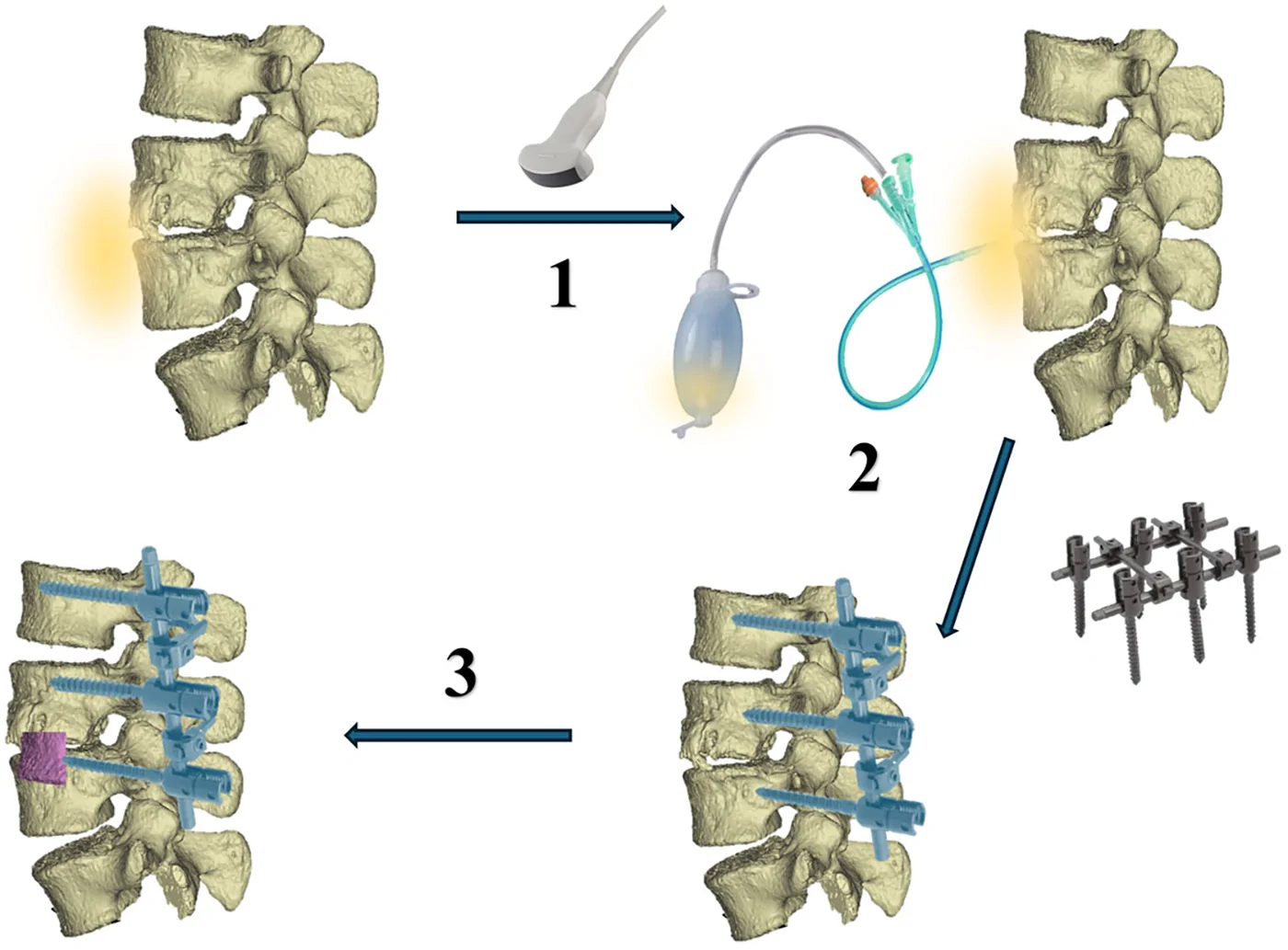
Individualized staged management pathway.

#### 2.5.2 Group B: combined posterior-and-anterior surgery

All patients underwent posterior pedicle screw fixation with or without spinal cord decompression and anterior radical debridement with interbody fusion. The patients were placed in the prone position for posterior fixation and decompression. Thereafter, the position was changed to the lateral position for anterior debridement and interbody fusion; therefore, the time spent repositioning and preparing for the anterior phase was included in the index operative time. In this group, paravertebral or psoas abscesses and anterior lesions were treated directly through the anterior surgical approach. Preoperative percutaneous drainage was not used because immediate anterior debridement and reconstruction were already indicated and provided direct abscess evacuation. The absence of preoperative drainage in Group B also illustrates that catheter drainage was not regarded as universally required. This study was not designed determine whether preoperative drainage is superior to direct drainage or debridement during definitive surgery. The detailed protocol for combined posterior and anterior approach surgery was described by Shen et al. (16). The operation process is illustrated in Figure 2. The study design is illustrated in Figure 3.

**Figure 2:**
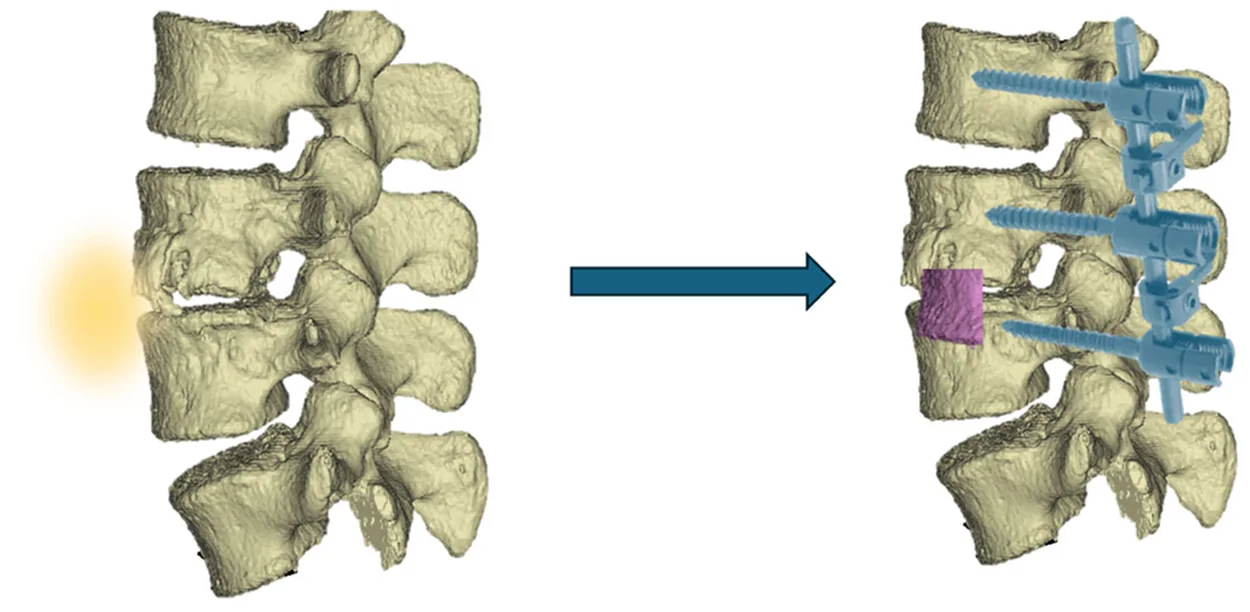
The process of combined posterior and anterior approach surgery management strategy.

**Figure 3:**
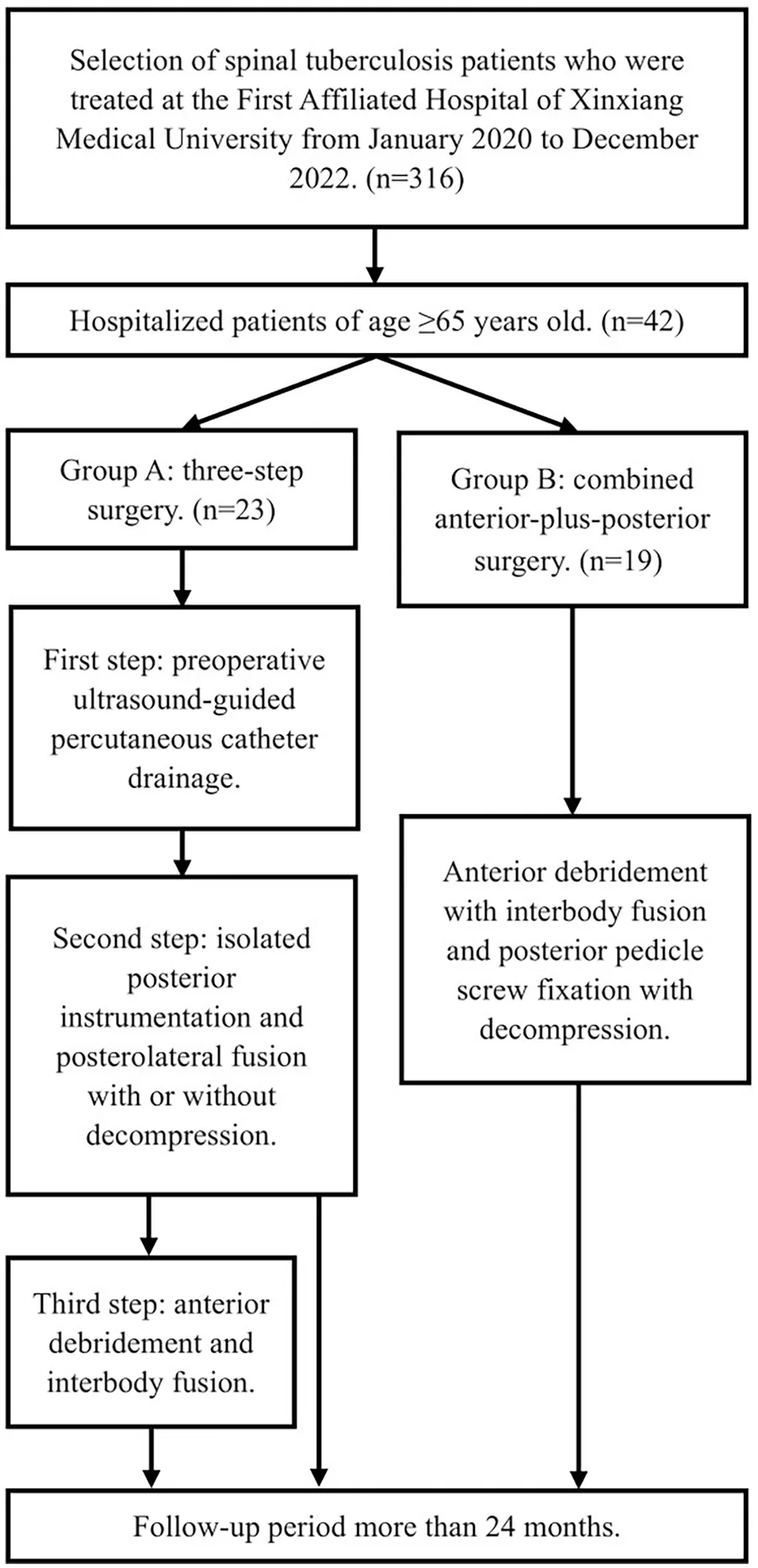
Study design.

### 2.6 Postoperative management and follow-up

Generally, chemotherapy with HRZE was continued for 12–15 months postoperatively. Anti-TB drugs were adjusted depending on the drug-sensitivity test results of each individual patient. Nutritional and support therapy were regularly enhanced. Every three to six months, radiography, CT, and MRI were performed. Surgical success was defined as clinical improvement, reduction of pain and disability, absence of major intraoperative complications, no need for unplanned reoperation for persistent infection, and radiographic evidence of healing or fusion, including trabecular bone bridging between the vertebrae and bone grafts when applicable.

### 2.7 Statistical analysis

Statistical summaries were generated using IBM SPSS Statistics (version 24.0; IBM, Chicago, IL, USA). Because the treatment pathways were defined by different clinical indications, the revised analysis is descriptive. Continuous variables are reported as mean ± standard deviation, categorical variables as number and percentage when applicable, and ordinal Frankel grades as counts. No inferential between-group testing was performed for baseline characteristics, index operative time, index EBL, transfusion variables, VAS, ODI, ESR, CRP, Cobb's angle, complications, recurrence, or secondary surgery. Frankel grades were also not tested statistically because of the small numbers in grades A–C. Accordingly, no distribution-dependent hypothesis test was applied to non-normally distributed continuous variables in the revised analysis.

## 3 Results

Presenting pain and neurological impairment are summarized in Table 1, and individual symptoms are provided in Supplementary Tables S1, S2. Forty-two patients were included: 11 male and 12 female patients in Group A (mean age, 71.65 years) and 10 male and 9 female patients in Group B (mean age, 69.84 years). Measured baseline characteristics are reported descriptively in Table 1 and are not used to establish clinical comparability because lesion-based indications and the need for immediate anterior surgery differed by design. Representative cases from Group A and Group B are shown in Figures 4, 5, respectively.

**Table 1:** Descriptive baseline characteristics of the two groups.

Baseline characteristics	Group A	Group B
Sex		
Female	12	9
Male	11	10
Age	71.65 ± 5.65	69.84 ± 4.00
BMI	23.89 ± 3.45	22.75 ± 1.81
Symptoms		
Pain	21	19
Neurological	6	5
Fever	7	5
Cough	2	1
Weight loss	10	8
Disease duration (months)	13.57 ± 27.55	5.18 ± 5.93
VAS	2.57 ± 0.59	2.47 ± 0.77
ODI	49.48 ± 10.11	48.42 ± 8.71
Frankel grading		
A	1	0
B	0	0
C	2	1
D	5	6
E	15	12
ESR	44.61 ± 3.46	41.26 ± 25.36
CRP	34.15 ± 54.81	30.11 ± 18.94
Cobb's angle	16.42 ± 9.00	19.17 ± 7.92
Segments		
1 vertebrae	1	0
2 vertebrae	20	19
3 vertebrae	2	0

BMI, body mass index; VAS, visual analog scale; ODI, Oswestry disability index; CRP, C-reaction protein; ESR, erythrocyte sedimentation rate.

Continuous variables are presented as mean ± standard deviation unless otherwise indicated. No inferential between-group testing was performed because pathway allocation was indication-based and the cohorts were clinically distinct.

**Figure 4:**
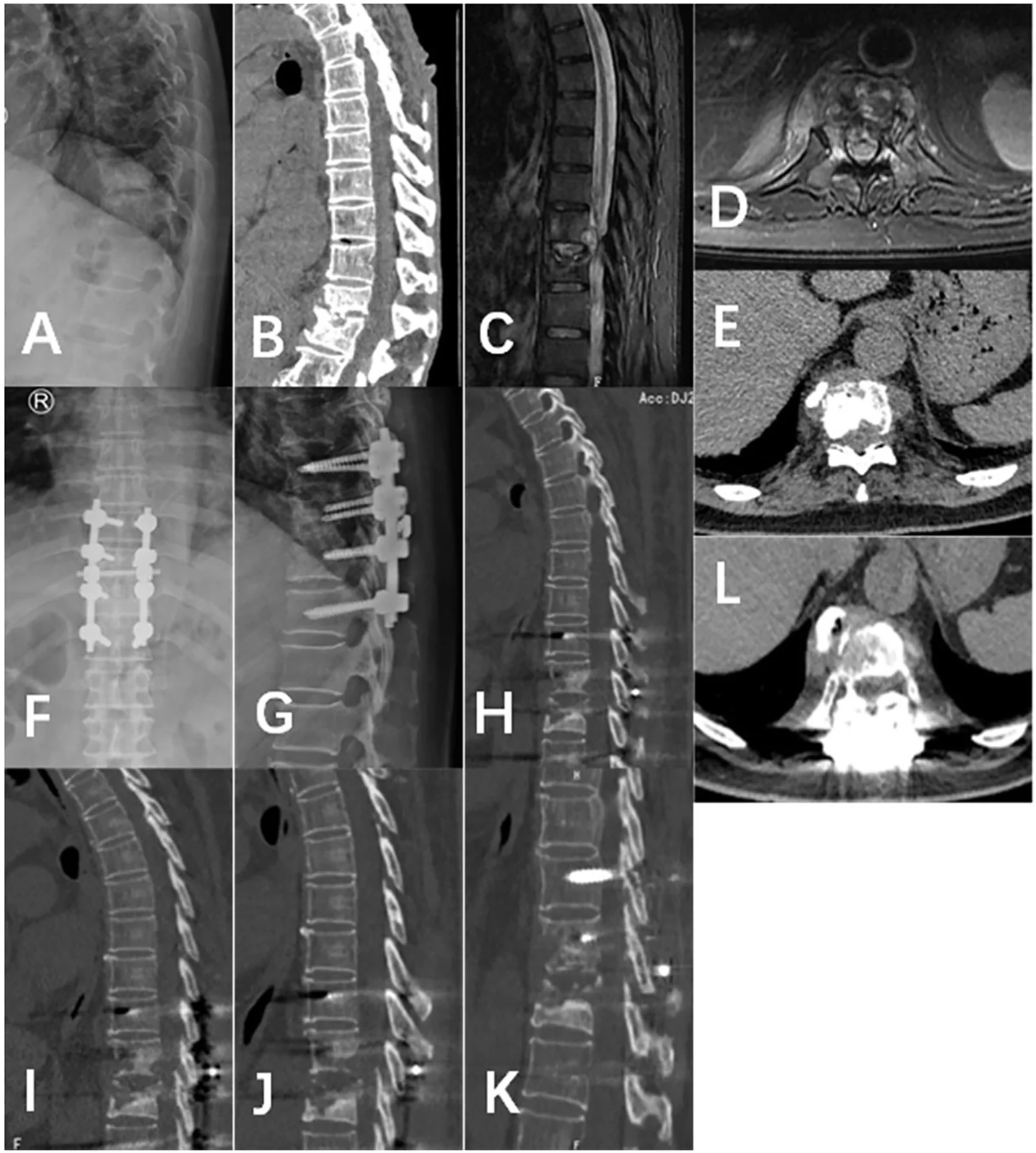
A 77-year-old male patient was diagnosed with thoracic tuberculosis at T10/11 and was treated using the individualized staged management pathway. **(A–E)**. Preoperative x-ray, CT, and MRI showing localized osteopenia, collapse of T11, abscess formation, and spinal cord compression. **(F,G)**. x-ray images 3 days after the definitive posterior operation showing satisfactory internal fixation. **(H–L)**. Follow-up CT at 5 days, 2 months, 5 months, and 15 months showed lesion control without obvious abscess, implant failure, or pedicle loosening.

**Figure 5:**
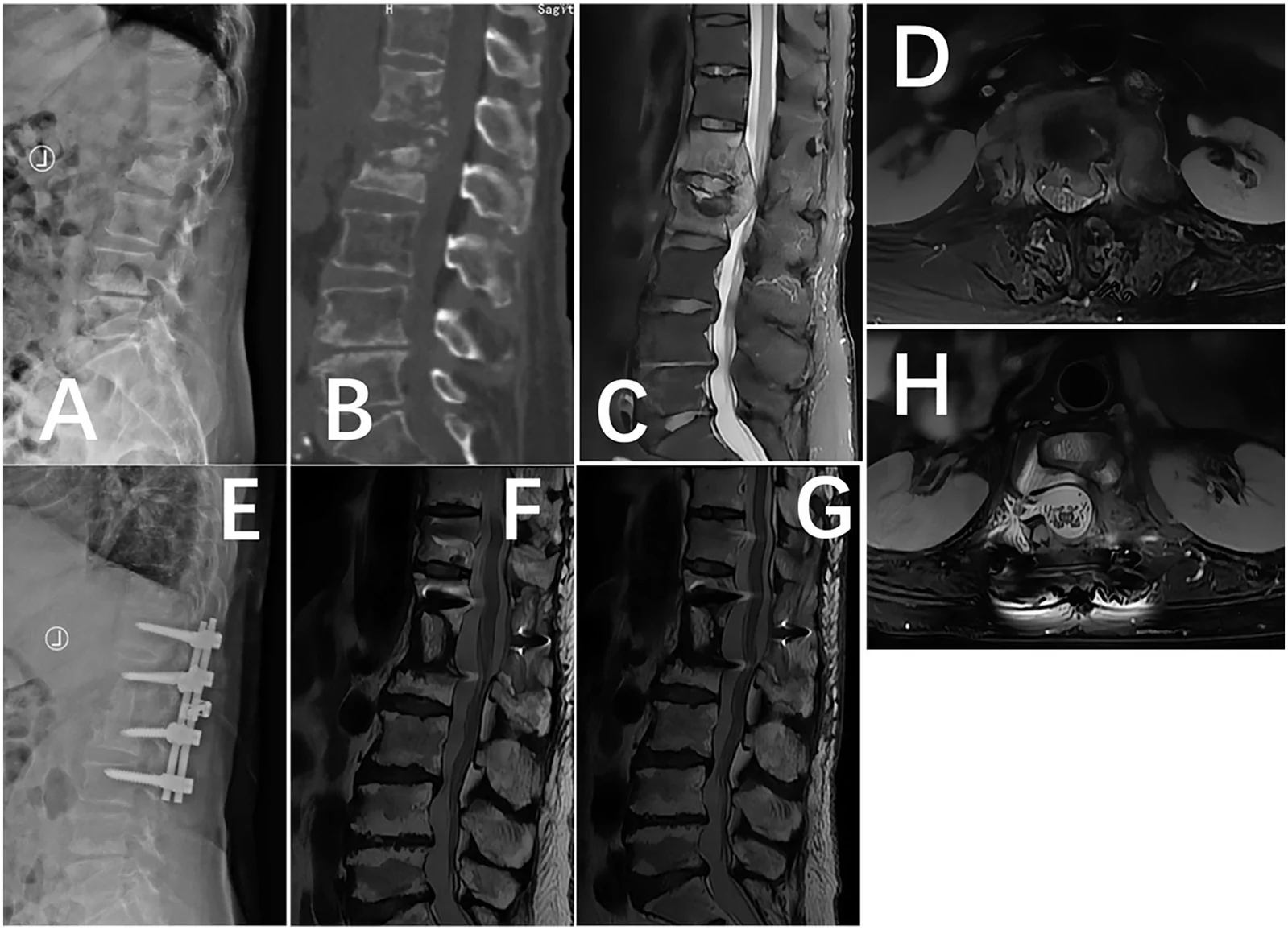
A 68-year-old female patient was diagnosed with lumbar tuberculosis in L1/2 and was treated with combined anterior plus posterior surgery. **(A–D)**. Preoperative x-ray image, CT and MRI showing localized osteopenia, collapse of the L2 vertebra, abscess formation and spinal cord compression. **(E)** The x-ray image 3 days after surgery showed good internal fixation and bone grafting. **(F)** Postoperative MRI performed at the 12-month follow-up showed no implant failure and pedicle loosening. **(G,H)**. Postoperative MRI performed at the 19-month follow-up showed complete debridement and no obvious abscess.

All planned index spinal procedures were completed without intraoperative death, major vascular injury, or anesthesia-related catastrophic complications. One patient in Group A developed a sinus two months after the index operation. Because infection control remained inadequate despite adjustment of the anti-TB regimen according to drug-sensitivity testing, wound care, and local drainage, the patient underwent salvage anterior debridement and interbody fusion. This later procedure was analyzed as a secondary operation and was not incorporated into the index operative time or EBL. The patients were followed for 24–45 months, with an overall average of 30.45 months. Mean index spinal operative time was 211.26 ± 70.30 min in Group A and 315.68 ± 70.92 min in Group B. Mean index EBL was 406.52 ± 224.26 mL and 594.74 ± 139.33 mL, respectively. Nine patients in each group received intraoperative blood transfusion, with mean transfusion volumes of 716.67 ± 351.78 mL in Group A and 833.33 ± 331.66 mL in Group B. Mean postoperative ODI was 17.22 ± 7.23 and 22.11 ± 3.97, respectively. By final follow-up, mean VAS, ODI, ESR, CRP, and Cobb's angle were lower than their corresponding baseline values in both groups (Tables 1, 2). These numerical contrasts are descriptive and reflect clinically selected pathways with different operative indications. The detailed surgical and postoperative characteristics are shown in Table 2.

**Table 2:** Descriptive index spinal surgical and postoperative characteristics of the two groups.

Variable	Group A	Group B
Index spinal operative time (min)	211.26 ± 70.30	315.68 ± 70.92
Index spinal estimated blood loss (mL)	406.52 ± 224.26	594.74 ± 139.33
Patients receiving intraoperative blood transfusion, *n* (%)	9 (39.13%)	9 (47.37%)
Blood transfusion volume (mL)	716.67 ± 351.78	833.33 ± 331.66
VAS		
Post-operation	2.83 ± 0.38	2.79 ± 0.53
Final follow-up	1.74 ± 0.61	1.89 ± 0.65
ESR		
Post-operation	47.04 ± 25.95	48.21 ± 20.45
Final follow-up	26.91 ± 21.46	33.73 ± 17.53
CRP		
Post-operation	54.37 ± 88.33	40.37 ± 35.26
Final follow-up	16.14 ± 13.17	23.65 ± 21.78
Cobb's angle		
Post-operation	9.18 ± 9.17	12.29 ± 7.91
Final follow-up	7.91 ± 7.38	10.37 ± 7.00
ODI		
Post-operation	17.22 ± 7.23	22.11 ± 3.97
Final follow-up	9.91 ± 4.68	8.95 ± 2.53

CRP, C-reaction protein; ESR, erythrocyte sedimentation rate; ODI, Oswestry disability index; VAS, visual analog scale.

Continuous variables are presented as mean ± standard deviation unless otherwise indicated. Values are descriptive; no inferential between-group testing was performed.

Frankel grade distributions shifted toward higher functional grades during follow-up (Table 3). In Group A, grades A–C accounted for 3 of 23 patients preoperatively and 1 of 23 at final follow-up, while grade E increased from 15/23 (65.2%) to 19/23 (82.6%). In Group B, grades A–C accounted for 1 of 19 patients preoperatively and 0 of 19 at final follow-up, while grade E increased from 12/19 (63.2%) to 16/19 (84.2%). Because of the small numbers in grades A–C and the descriptive study framing, no statistical test of Frankel grades was performed.

**Table 3:** Frankel grade distributions in the two groups.

Group	Time point	Frankel A	Frankel B	Frankel C	Frankel D	Frankel E
Group A	Preoperative	1	0	2	5	15
Group A	Final follow-up	0	1	0	3	19
Group B	Preoperative	0	0	1	6	12
Group B	Final follow-up	0	0	0	3	16

Values are counts; no inferential testing was performed because of sparse observations in grades A–C.

In Group A, 22 patients completed the drainage and posterior phases without anterior surgery. Recurrence occurred in one patient in Group A and three patients in Group B. One patient in each group underwent a secondary operation; the Group A procedure was the salvage anterior operation described above, whereas the Group B procedure was related to urinary TB. The detailed complication and secondary-surgery data are shown in Table 4. No postoperative pyogenic wound infection was observed in either group. The sinus formation and recurrence cases were considered related to persistent or recurrent TB infection. No additional bacterial pathogen was isolated from wound cultures. Mycobacterium tuberculosis was identified or clinically diagnosed based on microbiological, pathological, imaging, and laboratory findings.

**Table 4:** Complications and secondary surgery in the two groups.

Variable	Group A	Group B
Complications		
Wound infection		
Implant failure		
Sinus formation	1	
Recurrence	1	3
Pedicle loosening		
Neurological damage		
Secondary operation	1	1

Values are descriptive counts; no inferential between-group testing was performed.

## 4 Discussion

The principal conceptual issue in interpreting Group A is the role of preoperative ultrasound-guided catheter drainage. Our retrospective data do not establish that drainage was necessary before posterior surgery. Group B was treated without preliminary catheter drainage, and posterior surgery can itself provide decompression and drainage of accessible lesions when clinically appropriate. At our center, drainage-first treatment was used in neurologically stable Group A patients with accessible paravertebral or psoas abscesses as a preoperative adjunct intended to reduce the extra-spinal abscess burden before definitive posterior fixation. However, this sequence adds an intervention and may prolong treatment. Because the drainage-to-surgery interval, total hospital stay, and total treatment duration were not uniformly retrievable, we cannot determine whether reduction of abscess burden outweighed the added procedural and time burden. Ultrasound-guided drainage should therefore be interpreted as a selectable preoperative adjunct within this historical pathway, not as a mandatory stage or a procedure proven necessary by the present study.

The Group A findings are more appropriately interpreted in the context of posterior-based treatment literature than through comparison with Group B. Posterior decompression and instrumented fusion have been used for lumbar and lumbosacral spinal tuberculosis (5), while Lai et al. described preoperative percutaneous catheter drainage for lumbar spinal tuberculosis with psoas abscess (6). Kumar et al. also reported isolated posterior instrumentation in selected thoracolumbar spinal tuberculosis without routine anterior instrumentation or bone grafting (17). These reports support the clinical plausibility of posterior-based management with or without a preceding drainage procedure, although differences in patient selection, lesion severity, and reported endpoints preclude a numerical cross-study efficacy comparison. Combined posterior-and-anterior procedures remain established options when direct anterior debridement or reconstruction is required (16, 18, 19), and reports of complications associated with more extensive anterior or combined exposure provide additional context (20, 21). Age or frailty alone, however, should not be interpreted as an indication for catheter drainage.

Clinical and radiographic outcomes are therefore best considered as within-cohort descriptive trajectories. In both groups, mean VAS, ODI, ESR, CRP, and Cobb's angle were lower at final follow-up than at baseline, and the Frankel distributions shifted toward higher functional grades. These findings indicate improvement over follow-up in the treated cohorts, but they do not identify which component of treatment caused the change and do not demonstrate equivalence or superiority between the two pathways.

Within Group A, 22 of 23 patients completed drainage and posterior stabilization without salvage anterior surgery, whereas one patient required anterior debridement and interbody fusion two months later because of inadequate infection control. This observation supports feasibility of the intended pathway in the selected Group A cohort and shows that anterior surgery functioned as a contingency for patients initially judged not to require it; it does not imply that anterior surgery was unnecessary for Group B. Staged and combined procedures for spinal TB have been described previously (8, 9), so the contribution of the present study is the description of this indication-guided pathway in an elderly cohort rather than the invention of staging. A more direct evaluation of the drainage phase will require an indication-matched comparison between patients undergoing drainage followed by posterior surgery and patients undergoing posterior surgery without preceding catheter drainage, with total procedural burden and treatment duration prospectively captured.

Our study has several limitations. First, the sample size was small and follow-up was relatively limited. Second, the retrospective design precluded standardized prospective data collection and limited reconstruction of all clinical factors influencing pathway selection and timing. Third, the duration of ultrasound-guided drainage, EBL from the drainage procedure, the drainage-to-posterior-surgery interval, cumulative operative burden after salvage surgery, total hospital stay, and total treatment duration were not uniformly recorded. Therefore, the total burden of the drainage-first pathway cannot be compared with a single definitive operation. Fourth, the present dataset did not include an indication-matched posterior-only comparator specifically designed to evaluate whether preoperative drainage was necessary. Fifth, only one Group A patient required salvage anterior surgery, limiting conclusions regarding the optional anterior phase. Sixth, exclusion of cervical TB limits generalizability to that population. Neurological function was assessed using Frankel grading rather than the more widely used AIS because Frankel grades were consistently documented in the retrospective records. Finally, detailed follow-up data for conservatively treated patients were incomplete. The clinically distinct indications of Groups A and B are treated as a feature of this descriptive design rather than as evidence of comparability.

In conclusion, these data describe an individualized staged management pathway used in selected elderly patients with spinal TB whose lesions did not require immediate anterior debridement or reconstruction. Ultrasound-guided catheter drainage should be regarded as a selectable preoperative adjunct rather than a proven necessary stage. Combined posterior-and-anterior surgery remains appropriate when immediate anterior debridement or reconstruction is indicated. Future studies should directly compare drainage-plus-posterior surgery with indication-matched posterior surgery alone and should capture total procedural burden and treatment duration.

## Data Availability

The original contributions presented in the study are included in the article/Supplementary Material, further inquiries can be directed to the corresponding author.
