# Supplementary material for: Clinical outcomes of individualized surgical pathways in elderly patients with spinal tuberculosis: a retrospective cohort study in eastern China
Source: Front Med (Lausanne). 2026 Sep 21;13:1869144. doi: 10.3389/fmed.2026.1869144 (PMC13634970; doi:10.3389/fmed.2026.1869144)
Supplement: Supplementary file 1 [file Datasheet1.docx]

**Supplementary Table S1 Individual clinical characteristics of Group A**

| **Patient ID** | **Age (years) /sex** | **Symptoms** | **Segments** | **Comorbidity** |
| --- | --- | --- | --- | --- |
| 1 | 72/M | Back pain, Fever, Weight loss | L3-L4 | Osteoporosis |
| 2 | 73/F | Intermittent fever | L1-L2 | CHD |
| 3 | 74/M | Low back pain | T10-T11 | Osteoporosis, ISD |
| 4 | 77/M | Low back pain | T9-T10 | HBP, Osteoporosis, Obesity |
| 5 | 66/F | Low back pain, Radiating pain | L4 | HBP, DM, Obesity |
| 6 | 71/F | Low back pain, Weight loss | T12-L1 | ISD |
| 7 | 85/M | Low back pain, Weight loss, Cough, Fever | T12-L1 | COPD, ISD, Osteoporosis,  HBP, CI |
| 8 | 77/M | Low back pain, Radiating pain, Weight loss, Intermittent fever | L4-L5 | Osteoporosis, ISD |
| 9 | 73/F | Back pain, Weight loss | T7-T8 | Osteoporosis |
| 10 | 70/M | Back pain, Weight loss | T8-T9 | Obesity, CHD |
| 11 | 68/F | Back pain | T6-T7 | HBP, ISD |
| 12 | 77/F | Back pain, Weight loss | T7-T8 | Osteoporosis, ISD |
| 13 | 65/F | Low back pain | L3-L5 |  |
| 14 | 67/F | lower extremities weakness | T11-T12 | HBP |
| 15 | 78/F | Low back pain, Weight loss,  lower extremities weakness | T10-T11 | Osteoporosis, CHD |
| 16 | 82/M | Low back pain, Fever, Weight loss | T12-L1 | Osteoporosis, COPD |
| 17 | 65/F | Low back pain, Radiating pain | L3-L4 |  |
| 18 | 67/F | Low back pain, Cough | T8-T10 | HBP, ISD |
| 19 | 68/M | Radiating pain | L2-L3 | COPD, ISD |
| 20 | 71/M | Low back pain, Radiating pain | L1-L2 | CI, HBP, COPD |
| 21 | 65/M | Back pain | T10-T11 | CI, COPD |
| 22 | 72/M | Low back pain, Fever | L5-S1 | HBP, Osteoporosis, COPD |
| 23 | 65/F | Low back pain, Fever, Weight loss | T12-L1 | COPD |

**HBP**: high blood pressure; **DM**: diabetes mellitus; **CHD**: coronal heart disease; **COPD**: chronic obstructive pulmonary disease; **CI**: cerebral infarction; **ISD**: immune system disorder.

**Supplementary Table S2 Individual clinical characteristics of Group B**

| **Patient ID** | **Age (years)/sex** | **Symptoms** | **Segments** | **Comorbidity** |
| --- | --- | --- | --- | --- |
| 1 | 71/F | Weight loss, Back pain | T5-T6 | DM, Osteoporosis, COPD |
| 2 | 74/F | Low back pain, Radiating pain | L3-L4 | Osteoporosis, ISD |
| 3 | 68/F | Low back pain, Fever, Weight loss | L1-L2 | COPD, Obesity |
| 4 | 71/F | Low back pain, Weight loss | L1-L2 | COPD |
| 5 | 80/M | Low back pain, Fever | L4-L5 | HBP, CHD, CI, Osteoporosis |
| 6 | 67/M | Back pain, Weight loss | T12-L1 | DJD |
| 7 | 72/M | Low back pain, Cough | L4-L5 | HCV, Osteoporosis |
| 8 | 72/F | Low back pain, Fever | L1-L2 | DJD, Obesity |
| 9 | 72/M | Low back pain, Weight loss | L4-L5 | Osteoporosis |
| 10 | 69/M | Low back pain | T12-L1 | DM, COPD, CI |
| 11 | 65/M | Low back pain, lower extremities weakness | L4-L5 | COPD |
| 12 | 68/M | Low back pain, Weight loss | T11-T12 | PTB, COPD, ISD |
| 13 | 68/F | Low back pain, Radiating pain | T12-L1 | HBP, ISD |
| 14 | 66/M | Low back pain, Fever | L1-L2 | HBP, ISD |
| 15 | 65/M | Low back pain, Weight loss | L1-L2 | PTB, DJD, ISD |
| 16 | 71/F | Low back pain | L3-L4 | LDH, Osteoporosis, ISD |
| 17 | 67/M | Low back pain, Fever, Weight loss | T12-L1 | COPD |
| 18 | 76/F | Low back pain, Radiating pain | L5-S1 | Osteoporosis |
| 19 | 65/F | Low back pain, Radiating pain | L4-L5 | Osteoporosis |

**HBP**: high blood pressure; **DM**: diabetes mellitus; **CHD**: coronal heart disease; **COPD**: chronic obstructive pulmonary disease; **CI**: cerebral infarction; **ISD**: immune system disorder; **DJD**: degeneration of joint disease; **HCV**: hepatitis C virus; **PTB**: pulmonary tuberculosis; **LDH**: lumbar disc herniation.
